# A critical assessment for the value of markers to gate-out undesired events in HLA-peptide multimer staining protocols

**DOI:** 10.1186/1479-5876-9-108

**Published:** 2011-07-11

**Authors:** Sebastian Attig, Leah Price, Sylvia Janetzki, Michael Kalos, Michael Pride, Lisa McNeil, Tim Clay, Jianda Yuan, Kunle Odunsi, Axel Hoos, Pedro Romero, Cedrik M Britten

**Affiliations:** 1 Division of Translational and Experimental Oncology, Department of Internal Medicine III, University Medical Center of the Johannes Gutenberg-University, Mainz, Germany; 2 Department of Biostatistics, New York University, New York, NY USA; 3 ZellNet Consulting, Inc., Fort Lee, NJ USA; 4 Department of Pathology and Laboratory Medicine, University of Pennsylvania School of Medicine, Abramson Family Cancer Research Institute, Philadelphia, PA USA; 5 Vaccine Research East and Early Development, Pfizer Inc. Pearl River, NY USA; 6 Surgery and Immunology, Duke University Medical Center, Durham, NC, USA; 7 Ludwig Center for Cancer Immunotherapy, Memorial Sloan-Kettering Cancer Center, New York, NY USA; 8 Departments of Gynecologic Oncology and Immunology, Roswell Park Cancer Institute, Buffalo, NY, USA; 9 Bristol-Myers Squibb, Wallingford, CT USA; 10 Translational Tumor Immunology Group, Ludwig Center for Cancer Research of the University of Lausanne, Switzerland; 11 Research & Development, BioNTech AG, Mainz, Germany

## Abstract

**Background:**

The introduction of antibody markers to identify undesired cell populations in flow-cytometry based assays, so called DUMP channel markers, has become a practice in an increasing number of labs performing HLA-peptide multimer assays. However, the impact of the introduction of a DUMP channel in multimer assays has so far not been systematically investigated across a broad variety of protocols.

**Methods:**

The Cancer Research Institute's Cancer Immunotherapy Consortium (CRI-CIC) conducted a multimer proficiency panel with a specific focus on the impact of DUMP channel use. The panel design allowed individual laboratories to use their own protocol for thawing, staining, gating, and data analysis. Each experiment was performed twice and in parallel, with and without the application of a dump channel strategy.

**Results:**

The introduction of a DUMP channel is an effective measure to reduce the amount of non-specific MULTIMER binding to T cells. Beneficial effects for the use of a DUMP channel were observed across a wide range of individual laboratories and for all tested donor-antigen combinations. In 48% of experiments we observed a reduction of the background MULTIMER-binding. In this subgroup of experiments the median background reduction observed after introduction of a DUMP channel was 0.053%.

**Conclusions:**

We conclude that appropriate use of a DUMP channel can significantly reduce background staining across a large fraction of protocols and improve the ability to accurately detect and quantify the frequency of antigen-specific T cells by multimer reagents. Thus, use of a DUMP channel may become crucial for detecting low frequency antigen-specific immune responses. Further recommendations on assay performance and data presentation guidelines for publication of MULTIMER experimental data are provided.

## Background

Assays to evaluate antigen-specific immune response are increasingly used in cancer immunotherapy trials. The inherent complexity of T-cell assays has motivated several studies to address the harmonization and standardization of the most commonly used assays [1-8]. Since the introduction of HLA-peptide multimers (MULTIMERs) more than 15 years ago, the number of laboratories using these reagents to detect and quantify antigen-specific T cells has steadily increased, in part reflecting the high sensitivity and specificity of this assay platform [9]. The study described in this report is a continuation of a process actively pursued by the Cancer Research Institute's Cancer Immunotherapy Consortium (CRI-CIC) to develop comprehensive guidelines for harmonizing for MULTIMER experiments across laboratories. The first MULTIMER proficiency panel (MPP1) organized by CRI-CIC resulted in initial harmonization guidelines among which was the suggestion that use of a DUMP channel to exclude unwanted cells carrying surface markers (such as CD4, CD14 or CD19) might be a critical factor determining test performance [7]. Since the addition of antibody markers increases the complexity and costs of the assay, it is important to demonstrate that this additional effort provides clear benefit in terms of assay performance and data quality.

Here we present the results of a second MULTIMER proficiency panel to systematically evaluate, for the first time, the effect of DUMP channel markers on MULTIMER assay performance across individual laboratory protocols. PBMC samples from four preselected donors with well defined numbers of antigen specific CD8^+ ^T cells were distributed to participating labs from a central facility. The panel design allowed all labs to use their own protocol for thawing, staining, gating, and data analysis. Each laboratory performed two parallel assays, one with and one without the inclusion of dump channel markers.

The study revealed a clear benefit for the use of a DUMP channel, extending the observations from the initial proficiency panels. The benefit for applying dump channel strategies was apparent in a large fraction of independent experiments across multiple laboratories and using independent staining, acquisition, gating and analysis protocols. Finally, new recommendations on how to best display results from MUTIMER staining are given.

## Methods

### Panel design and organizational setup

The second MULTIMER proficiency panel was conducted with a group of 20 centers. Participating laboratories were located in seven countries (Belgium, Canada, Germany, Japan, Sweden, Switzerland and USA). Organizational and scientific panel leadership was provided by two leaders experienced in MULTIMER staining, in collaboration with the CIC executive office and the steering committee of the CIC Immunoassay working group. The authors of this group acknowledge the concept of the Minimal Information About T cell Assays (MIATA) reporting framework for human T cell assays that was recently introduced to the community [10,11]. Consequently, we provide structured information on 5 modules: the sample, the assay, the data acquisition, the data analysis and interpretation and finally, the lab environment in which the corresponding T cell experiments were performed.

### The sample

Four healthy donors provided written informed consent for this study prior to a leucapheresis. PBMC were obtained from the Immunology Quality Assurance Center Laboratory (IQAC) of the Duke Human Vaccine Institute, a division of the Duke University Medical Center in Durham NC. Samples were obtained via leukapheresis and processed in the IQAC laboratory within 4 hours of collection. PBMC were separated by density gradient centrifugation, cryo-preserved in 10% DMSO and 90% heat-inactivated FBS at 15 million cells per vial using an automated controlled rate freezer, and stored in equal aliquots in two vapor phase LN2 freezers.

Pre-screening to identify donors with peripheral CD8+ T cells specific for HLA-A*0201-restricted epitopes from CMV pp65_495-503 _(NLVPMVATV) and Melan-A/Mart-1_26-35 _(ELAGIGILTV) was conducted at the Lausanne branch of the Ludwig Institute for Cancer Research (LICRLB). Donor selection was based on evaluation using three different sources of MULTIMERs; donor samples were identified that had antigen-specific CD8^+ ^T cells at a frequency of ≤ 1 in 500.

For this study PBMC from four HLA-A*0201 donors were selected; 3 donors (D1, D3, D4) were CMV seropositive while D2 was CMV seronegative; since D2 did not contain detectable levels of CMV pp65-specific T cells this sample was used as a negative control for these analyses (Additional file 1, Figure S1). Each participating laboratory received 2 vials from each donor, each vial containing 15 × 10^6 ^PBMCs. Participating labs were asked to store the samples in liquid nitrogen upon arrival. The method used for thawing and counting of vials was left to the discretion of the participating labs. The total cell number after thawing and the number of viable cells were documented and reported in a questionnaire. The mean cell viability of cell material was 86% with similar results for all 4 donors. Under optimal conditions, a participating lab should have identified a population of CMV pp65- or Melan-A-specific CD8^+ ^lymphocytes in seven donor-antigen combinations. Donor 2 did not contain detectable levels of CMV pp65-specific T cells and can be regarded as a negative control (Additional file 1, Figure S1).

### HLA-peptide multimer staining

Participants were free to choose HLA-peptide tetramers or pentamers. The MULTIMERS were generously donated by Beckman Coulter (Fullerton, CA) or ProImmune (Oxford, UK), respectively. Sixteen laboratories used HLA-peptide tetramers and 6 laboratories used HLA-peptide pentamers. Each lab received one vial of the MULTIMER specific for i) a defined and unknown peptide sequence (irrelevant multimer), ii) CMV pp65_495-503 _(Antigen "A1" = NLVPMVATV) and iii) Melan-A/Mart-1_26-35 _(Antigen "A2" = ELAGIGILTV). Each of the participating laboratories were required to use 10 μl per staining of a given MULTIMER.

Individual laboratories used different methods to count viable cells, their own staining protocols and were free to choose all other parameters such as buffers, serum supplement, plates, tubes, staining volume, incubation time and the inclusion of a dead cell marker. Staining was done in duplicate, for two different conditions (once with and once without utilizing dump channel markers), otherwise following the same laboratory-specific protocol. Six stainings were requested for each donor and condition (+/- dump channel): an FMO staining, a staining with irrelevant MULTIMER, duplicate stainings with the CMV and Melan-A multimers. The staining with the irrelevant MULTIMER was used as a negative control. At least 2 different cell surface antigens had to be used for the dump channel, with one being CD19. All other antigen choices (e.g. CD4, CD13, CD56 etc.) were left to the discretion of the lab.

### Data acquisition

Individual laboratories acquired the data on their flow-cytometer and analyzed the FCS files following laboratory-specific analysis strategies and software. The requested format for presenting the results was a series of plots showing CD8 on the x-axis and the MULTIMER on the y-axis. Participants were explicitly asked to count at least 100,000 CD8-positive events, based on previous panel findings and initial harmonization guidelines [7]. Representative dot plots from all participating labs will be made available upon request.

### Data Analysis and Interpretation

#### Data generated by individual laboratories were evaluated in 2 ways

Initial analysis was performed in a non-censored manner using the numerical data generated and provided by individual laboratories. In addition, to minimize the impact of individual laboratory gating, analysis, and interpretation strategies, a censored analysis was also performed. For the censored analysis, three criteria were applied to determine if an individual lab successfully detected a response; these criteria required (i) a reproducible duplicate staining and (ii) the presence of a clearly clustered population of MULTIMER-positive CD8^+ ^cells as assessed by an visual inspection of the dot plots during an independent central assessment and (iii) a reported value of less than 1% of MULTIMER-positive CD8^+ ^cells. Stainings for each multimer/donor combination were considered reproducible if the percentage difference between the two replicate measurements was less than 200%. Since the definition of a "clearly clustered population" is subjective in nature, two experienced evaluators independently examined each the dot plots and assigned a score based on whether there was a clustered population. A score of 0 was given when there was no obvious clustering ("clearly negative") or the experiment was not performed or the dot plot appearance was ambiguous ("unclear"), a score of 1 was given for ambiguous results, and a score of 2 was given when there was a clustered population of dots ("clearly positive"). Consequently, each duplicate staining could reach scores ranging from 0 to 4. A score greater than two was considered as evidence of a clearly clustered population of MULTIMER^+ ^CD8^+ ^cells. A laboratory was deemed to have detected a response if both criteria (acceptable reproducibility between duplicate measures and presence of clearly clustered multimer^+ ^population) were met. Four individual experiments were excluded even though they met both criteria due to the fact that the frequencies of antigen-specific CD8^+ ^T cells for these experiments were > 1%, a 5-fold higher value than the highest frequency as determined during pretesting by the central laboratory ("completely out of range").

### Statistical Methods

The following parameters were calculated for the overall panel performance using the lab-specific reported percentage of MULTIMER^+ ^CD8^+ ^cells: the median percentage of CD8^+ ^cells for each donor and antigen and the coefficient of variation (CV). To compare the percentage of MULTIMER^+ ^CD8^+ ^cells reported between experiments performed WITH a dump channel versus NO dump channel and between experiments that were analysed centrally using different gating strategies, the Wilcoxon signed rank test for paired comparisons was used. To compare the percentage of MULTIMER^+ ^CD8^+ ^cells between labs that used different gating strategies, the two sample Wilcoxon test was used. The association between non-specific and specific MULTIMER binding (percentage of MULTIMER^+ ^CD8^- ^cells versus percentage of MULTIMER^+ ^CD8^+ ^cells) was assessed with Spearman's correlation coefficient.

### Lab environment

Participating laboratories operated under different principles, varying from exploratory research to Good Laboratory Practice (GLP). All labs followed their own, previously established protocols. There were large differences in the experience level of the operator as reported by the participants. Ten labs reported more than 3 years of experience in the use of the technique whereas 10 labs reported less than two years of experience.

## Results

### Quality of experimental data

MULTIMER experiments should be conducted with cell material of high viability [12] and be based on sufficient cell counts [7,13]. In order to obtain evidence that cell material of sufficient quality and quantity was used in the second MULTIMER panel all participants were asked to record cell viability for each donor. Cell viability as determined by trypan blue exclusion was excellent, with a mean viability of 85, 89, 86 and 85% for donors D1 to D4 respectively (Table 1).

**Table 1:** Cell Viability

Viability (%)	Donor	Mean	Median	< 70%	70-100%
	1	84.7	86.2	3 (15%)	17 (85%)
	2	88.5	90.5	1 (5%)	19 (95%)
	3	86.3	86.1	0 (0%)	20 (100%)
	4	85.0	87.2	2 (10%)	18 (90%)

The table reports the overall viability for each of the thawed PBMC donor samples as determined by trypan blue staining. The table presents the mean and median viability for each donor. It also reports the proportion within optimal and suboptimal ranges.

Laboratories were further required to report the number of acquired CD8^+ ^events. The median CD8^+ ^event counts were > 79,000 in D2, > 95,000 in D4 and D3 and > 100,000 in D1. Further, the median event counts derived from both conditions (with and without DUMP channel) for any of the four donors were similar (Table 2).

**Table 2:** CD8-positive event counts

Event count	Donor	Dump Channel	Median	Mean
	1	No	101983	123825
		Yes	105629	116992
	
	2	No	79964	82570
		Yes	80243	81993
	
	3	No	101239	118428
		Yes	99947	110498
	
	4	No	100732	103625
		Yes	95015	94656

The table shows the range of events counted in the conditions stained with the CMV-pp65 MULTIMER for all four donors.

### Introduction of a DUMP channel decreases the amount of non-specific events observed in the CD8-positive cell fraction

The main aim of this proficiency panel was to systemically study the impact of DUMP channel use across representative assay protocols. To this end each participant performed paired sets of experiments that only differed in the use of a DUMP channel. All other assay variables were kept constant.

#### Non-censored analyses

**Figure 1:**
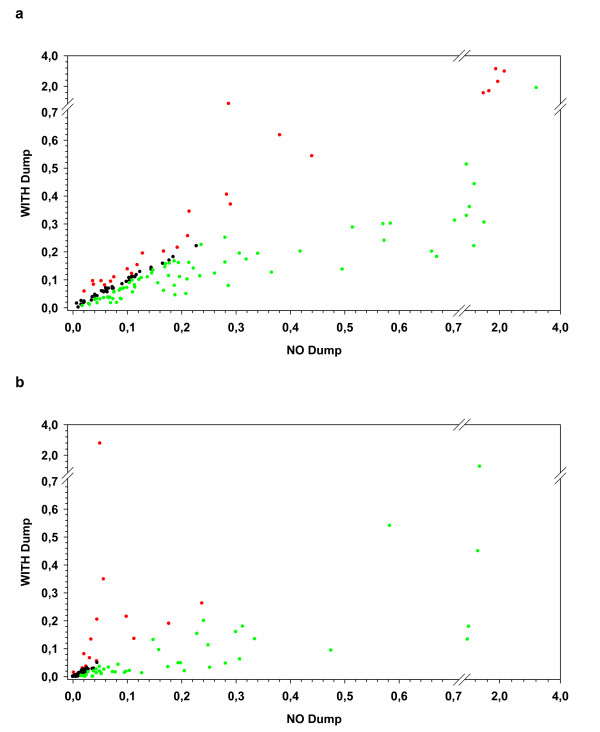
**MULTIMER binding in the condition WITHOUT versus WITH use of a DUMP channel**. The figure shows results for the percentage of MULTIMER-positive CD8-positive events in the condition WITHOUT DUMP (x-axis) and WITH DUMP (y-axis) for (a) the seven positive donor-antigen combinations after staining with the CMV- or Melan-A MULTIMER and (b) the negative donor antigen combination (CMV in D2) as well as the results generated when using the irrelevant MULTIMERS (D1 to D4). Experiments with an increase (> 0.01%) of non-specific MUTIMER binding in the condition with DUMP are shown in red. Experiments with a decrease (> 0.01%) of non-specific MULTIMER binding in the condition with DUMP are shown in green.

A comparison within each lab was made between the MULTIMER^+ ^CD8^+ ^events reported in the experiments WITH DUMP versus WITHOUT DUMP channel markers. Figure 1a displays these paired experiments for all seven donor-antigen combinations where a response was expected. The WITHOUT DUMP results are presented on the x-axis and the results WITH DUMP on the y-axis. In total a 1.65-fold reduction of background was observed across all experiments with irrelevant MULTIMERs. Three classes of experimental outcomes were observed with regard to the quantification of MULTIMER^+ ^CD8^+ ^events. In the largest fraction of experiments (53.6%) a decrease of non-specific MULTIMER binding (median -0.055%) was observed in the condition WITH DUMP channel. In a small fraction (17.9%) of paired replicates we observed an increase of MULTIMER-positive CD8^+ ^events in the condition WITH DUMP channel (median increase 0.045%). In a third fraction (28.5%) of paired replicates there were similar results obtained for both conditions (difference < 0.01%). Examining the median reported % MULTIMER^+ ^CD8^+ ^events for each donor and reagent and experimental condition including all reported data sets, it is apparent that the results from the WITH DUMP channel experiments on average led to lower values than the results from the NO DUMP channel experiments in all eight tested donor-antigen combinations (Table 3).

**Table 3:** %age of CMV pp65- and Melan-A-MULTIMER-positive CD8-positive events

MULTIMER	Donor	Dump Channel	**Median***(raw)*	
CMV pp65	1	No	0.12	↓
		Yes	0.10	
	
	2	No	0.04*	↓
		Yes	0.02*	
	
	3	No	0.17	↓
		Yes	0.14	
	
	4	No	0.08	↓
		Yes	0.07	

Melan-A	1	No	0.17	↓
		Yes	0.09	
	
	2	No	0.24	↓
		Yes	0.18	
	
	3	No	0.10	↓
		Yes	0.08	
	
	4	No	0.06	↓
		Yes	0.04	

The medians of the reported percentages of MULTIMER-positive CD8-positive cells for each antigen-donor combination are shown in the table. These results are stratified by condition (with and without the inclusion of a dump channel). Results obtained using two MULTIMERS in four donors stratified by use of a DUMP channel. For all sixteen experimental conditions the median of the reported values for MULTIMER+ CD8+ cells for all experiments are displayed. The asterisk indicates a negative control donor.

MULTIMER^+ ^CD8^+ ^events can either result from specific MULTIMER binding to antigen-specific TCRs (true specific signal) or from non-specific binding of MULTIMER to lymphocytes (non-specific signal). To address the question of whether the reduction of MULTIMER^+ ^CD8^+ ^events was due to loss of true specific signal or reduction of non-specific signal we focused on results obtained with the irrelevant MULTIMER. Here we assume that all MULTIMER^+ ^CD8^+ ^events must result from non-specific MULTIMER binding.

When focusing on the paired replicates generated with the irrelevant MULTIMER and the CMV MULTIMER in the CMV-negative donor D2 we identified three classes of experimental outcomes (Figure 1b). In the largest fraction of experiments (48 of 100) we found a decrease of non-specific MULTIMER binding (median -0.049%) in the condition WITH DUMP (green data points) which represents a 4.1-fold median reduction of the background staining in this subgroup of experiments. Interestingly, this group included 31 experiments in which use of a DUMP channel was combined with a dead cell dye, showing that in a large fraction of representative protocols the addition of a DUMP channel to a dead cell dye may have favourable effects. In a small fraction (15 of 100) of paired replicates we observed an increase of MULTIMER^+ ^CD8^+ ^events in the condition WITH DUMP (median increase 0.035%) (red data points). In a larger fraction (37 of 100) of paired replicates there were similar results obtained for both conditions (difference < 0.01%) (black data points); thirty one of these 37 experiments included the use of a dead cell dye.

Table 4 displays the median frequency of MULTIMER^+ ^CD8^+ ^cells after applying the irrelevant MULTIMER for both conditions stratified by the use of dead cell staining. Comparison of the amount of irrelevant MULTIMER binding showed that the median difference between WITH DUMP and NO DUMP for the paired replicates from labs that did not use a dead cell marker was 0.02% (Table 2). The median difference for the paired replicates from labs that did use a dead cell marker was only 0.01%. Therefore those labs that did not use a dead cell marker, on average measured a larger reduction of non-specific MUTLIMER staining after addition of a DUMP channel.

**Table 4:** %age of Irrelevant-MULTIMER-positive CD8-positive events

MULTIMER	Donor	Dump Channel	Dead Cell Staining	N	Median	
Irrelevant	1	No	No	6	0.04	↓
		No	Yes	14	0.02	
		Yes	No	6	0.04	↓
		Yes	Yes	14	0.01	
	
	2	No	No	6	0.06	↓
		No	Yes	14	0.03	
		Yes	No	6	0.05	↓
		Yes	Yes	14	0.02	
	
	3	No	No	6	0.04	↓
		No	Yes	14	0.03	
		Yes	No	6	0.02	↓
		Yes	Yes	14	0.02	
	
	4	No	No	6	0.03	↓
		No	Yes	14	0.02	
		Yes	No	6	0.03	↓
		Yes	Yes	14	0.01	

Results obtained using the irrelevant MULTIMERS in four donors stratified by DUMP channel use and further subdivision by the use of dead cell marker. The table also indicates the number of labs (N) for each of the 16 subgroups. The table further indicates the median values of the reported percentages of MULTIMER+ CD8+ cells for all reported data sets using the irrelevant MULTIMER. Arrows in both tables denote decreased values when a DUMP channel is used.

#### Censored analyses

Upon central review of all data sets from this second proficiency panel, it became clear that the reported results contained (i) duplicate stainings with discordant results, (ii) dot plots devoid of a clear clustered MULTIMER^+ ^CD8^+ ^population for the donor-antigen combinations expected to be positive and (iii) a reported frequency of MULTIMER^+ ^CD8^+ ^T cells far above 1%, which is more than 5-fold above the expected maximum value of 0.2% and therefore are clear outliers. Since such inconsistencies in the submitted data sets might influence the clear effects seen for introduction of a DUMP channel we applied three intuitive data filters to determine if a given staining should indeed be considered a successfully detected response.

The first criterion selected for reproducible duplicate values (Table 5). Discordant duplicates defined as percent difference greater than 200%, were not considered a positive response. Thirty nine replicates (12%) with high variation between the duplicate measurements fell into this group.

**Table 5:** Data Filter 1 - Reproducibility

			Percent Difference between Duplicates			
Antigen	Donor	Dump Channel	0-10%	10-30%	30-200%	> 200%*
CMV p65	1	No	9	3	5	**3**
		Yes	9	5	3	**3**
	
	2	No	6	6	4	**4**
		Yes	4	5	3	**8**
	
	3	No	13	5	2	**0**
		Yes	9	9	2	**0**
	
	4	No	9	9	1	**1**
		Yes	3	10	6	**1**

Melan-A	1	No	8	3	7	**2**
		Yes	6	11	3	**0**
	
	2	No	7	5	6	**2**
		Yes	7	8	5	**0**
	
	3	No	7	7	5	**1**
		Yes	5	10	2	**3**
	
	4	No	8	4	2	**6**
		Yes	5	5	5	**5**

Filter 1: Reproducibility, Based on Percent Difference. The datasets were grouped by the variation of reported MULITMER-positive frequencies in staining duplicates. Duplicates that showed high variation (> 200%) were not considered as a positive response and are indicated in bold. *This group also includes duplicates with missing data, namely only one staining was performed.

The second criterion was a visual inspection of the dot plots to determine if the dot plot showed a clear clustered population of MULTIMER^+ ^CD8^+ ^cells. The scores assigned by two independent evaluators for each dot plot were compared. In case of disagreement, a consensus score was agreed upon by both evaluators: there were only 11 instances of initial discordance. The sum of the dot plot scores for each staining in a duplicate was calculated and experiments with duplicates that had a total score of ≤ 2 were not considered a positive response. These are indicated in bold in Table 6. A total of 132 replicates (41%) fell into this group.

**Table 6:** Data Filter 2 - Visual Confirmation

			Sum of Dot Plot Evaluation Score*				
Antigen	Donor	Dump Channel	0	1	2	3	4
CMV p65	1	No	**0**	**0**	**1**	3	16
		Yes	**0**	**0**	**2**	3	16
	
	2	No	**20**	**0**	**0**	4	0
		Yes	**19**	**1**	**0**	8	0
	
	3	No	**0**	**0**	**1**	0	19
		Yes	**0**	**0**	**0**	0	20
	
	4	No	**0**	**1**	**1**	1	16
		Yes	**0**	**1**	**1**	1	17

Melan-A	1	No	**3**	**2**	**5**	2	10
		Yes	**3**	**1**	**4**	0	12
	
	2	No	**4**	**2**	**5**	2	8
		Yes	**4**	**0**	**4**	0	12
	
	3	No	**5**	**1**	**4**	1	9
		Yes	**1**	**2**	**5**	3	11
	
	4	No	**8**	**3**	**3**	6	6
		Yes	**7**	**1**	**7**	5	4

Filter 2: Visual Confirmation from Dot Plot Evaluation. The reported dot plots were assessed by a central review of all the dot plots. A dot plot was assigned a score of "0" when there was clearly no clustered population (or the experiment was not performed or not interpretable), a score of "1" when the clustering was ambiguous and a score of "2" when there was clearly a clustered population. The sum of the scores for each duplicate is presented in the table. The columns in bold indicate experiments that did not meet the optical evaluation criteria (< = 2) and therefore were not considered a positive response.

The visual inspection of dot plots is an intuitive and subjective method for evaluating response detection employed routinely by laboratories performing a MULTIMER assay. The unexpected high fraction of results (41% of all dot plots) that did not pass our strict filter criteria stimulated us to check whether the dot plot scores generated by the central reviewers overlaps with the judgement of the individual investigators that had to record whether they consider any given staining with one of the two-relevant MULTIMERS as a successfully detected response (yes/no). Interestingly, clear disagreement between the central evaluation and the lab evaluation was only observed in 12% of all experiments (74/636 stainings) and was equally distributed between the pp65 MULTIMER (12% clear disagreement) and the Melan-A MULTIMER (11% clear disagreement; Additional file 1, Table S1).

The third filter applied was plausibility and called for exclusion of MULTIMER positive values greater than one percent. There were a total of 38 stainings that resulted in greater than 1% MULTIMER specific binding with 35 (92%) of these outlier values reported by three labs (ID13, ID18 and ID19) suggesting technical difficulties. Any duplicate where one or both of the stainings were greater than 1% did not meet this criterion resulting in 21 replicates not being considered a positive response. In fact, only 4 of these 21 replicates passed both of the first two criteria. The reason for the outlying event counts in the upper right quadrant for these four duplicates were large MULTIMER^dim ^CD8^dim ^population of cells in three cases and one dot plot in which a large MULTIMER^dim ^population occurred in the CD8-positive cells (not shown).

Applying these three filters allowed us to test whether the favourable effects of DUMP channel that were observed examining all the data sets could also be observed after eliminating experiments that could contain potential artefacts and hence would not be considered to have detected a response. Table 7 shows the median frequency of reported antigen-specific T cells response and the detection rates for all donor antigen combinations for both conditions. When focusing only on those paired experiments (N = 78) that passed all three filters for both conditions (DUMP and NO DUMP), WITH dump channel results in all donor-antigen combinations were on average lower than NO dump channel results (Median difference: 0.01, 95% CI: 0.01, 0.02, p < 0.001 Wilcoxon signed rank test). The majority of labs were able to successfully detect (passed all three filters) the three low pp65-specific T cell responses. Interestingly, the detection rates for experiments with the Melan-A MULTIMER were much lower than for pp65 MULTIMER although responses against both antigens were similar in frequency across the four donors. Comparing the response detection rates between the two conditions it appears that including a DUMP channel did not lead to a higher detection rate.

**Table 7:** Filtered Dataset and Detection Rate

MULTIMER	Donor	Dump Channel	Median *(filtered)*	Detection Rate
CMV pp65	1	No	0.11	16 (80%)
		Yes	0.10	17 (85%)
	
	2*	No	n.a.	0 (0%)
		Yes	n.a.	0 (0%)
	
	3	No	0.17	18 (90%)
		Yes	0.14	20 (100%)
	
	4	No	0.08	17 (85%)
		Yes	0.06	18 (90%)

Melan-A	1	No	0.18	10 (50%)
		Yes	0.16	12 (60%)
	
	2	No	0.23	9 (45%)
		Yes	0.18	12 (60%)
	
	3	No	0.10	10 (50%)
		Yes	0.09	10 (50%)
	
	4	No	0.06	5 (25%)
		Yes	0.04	5 (25%)

Filtered results obtained using two MULTIMERS in four donors stratified by use of a DUMP channel. For all sixteen experimental conditions the (i) the median of the reported values from experiments with a positive response in both conditions (filtered), and (ii) response detection rates are displayed. The asterisk indicates a negative control donor.

### *In silico *study on the independent value of DUMP channel markers and dead cell dye use

**Figure 2:**
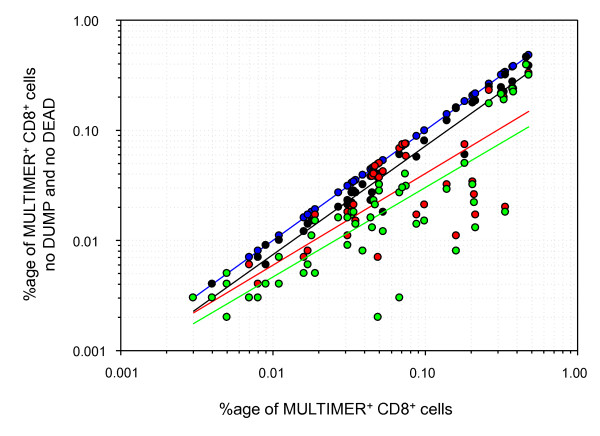
***In silico* study**: The figure shows the frequency of events detected in the MULTIMER-positive CD8-positive fraction when neither DUMP channel markers nor dead cell dyes were included in the gating strategy (x-axis) and the four corresponding event counts on the y-axis in the gating strategy NO DEAD and NO DUMP (blue), WITH DEAD and NO DUMP (black), NO DEAD and WITH DUMP (red), WITH DEAD and WITH DUMP (green). The figure also shows the resulting linear regression curves for each of the four data sets.

In order to determine the relative impact of DUMP channel markers and/or dead cell dye use to reduce the background signal in MULTIMER experiments an *in silico *study was performed. To this end, available FCS files from this proficiency panel phase that originated from the seven participating centers that applied both a dead cell dye and DUMP channel markers were revisited. A total number of 53 available FCS files representing stainings performed with the irrelevant MULTIMER and the CMV-multimer in CMV-negative donor D2 were re-analyzed using four different gating strategies for each file (NO DUMP/NO DEAD and NO DUMP/WITH DEAD and WITH DUMP/NO DEAD and WITH DUMP/WITH DEAD). As shown in Figure 2 the highest signals were typically observed when NO DUMP and NO dead cell dye were applied in the gating strategy (blue). Excluding dead cells led to a decrease of the non-specific signal (black) in a large fraction of experiments which was even higher when DUMP channel markers were included (red) in the gating strategy and highest when a dead cell dye and DUMP were combined (green). The median values observed for the four different gating strategies as mentioned above were 0.046% (NO DUMP/NO dead cell dye), 0.027% (NO DUMP/WITH dead cell dye), 0.018% (WITH DUMP/NO dead cell dye) and 0.015% (WITH DUMP/WITH dead cell dye), respectively. The use of DUMP channel markers or dead cell dye or the combination of both lead to a significant reduction (Wilcoxon rank sum test; p < 0.001 in all three tests) of the non-specific signal compared to the results obtained without gating out unwanted cells. In addition the combination of DUMP channel markers and a dead cell dye led to a significant reduction compared to the use of either DUMP channel markers or dead cell dye alone (Wilcoxon rank sum test; p < 0.001).

Interestingly, the median decreases between the four different gating strategies in the *in silico *study matched the results that were observed when comparing results generated by the different labs and staining conditions.

### Influence of gating styles and role of MULTIMER binding to CD8-negative cells

A well-known critical factor in determining the amount of antigen specific cells is the placement of gates and/or quadrants. Central review of the dot plots revealed that about 12 from 20 participating labs placed the upper right gate close to the antigen negative population ("CLOSE" gating style) whereas 6 of the 20 labs placed the horizontal gate in such a way that it was quite distant from the MULTIMER-negative population of events ("DISTANT" gating style; see inserted dot plots adjacent to Table 8). Two labs applied a mixed gating style with some gates being close to and some distant from the MULTIMER-negative population. The 18 participants with consistent gating style were stratified in two subgroups (CLOSE vs. DISTANT) and the median event counts in the upper right quadrant for the two relevant MULTIMERS (pp65 and Melan-A) are displayed in Table 8. There were significant differences in the frequencies of pp65- (p < 0.001, two sample Wilcoxon test) and Melan-A-specific (p < 0.001, two sample Wilcoxon test) cells for close or distant gating strategies, with close gating leading to much larger reported percentages of CD8+ MULTIMER positive cells than distant gating. The difference in the median percentages of CMV pp65-specific cells between close and distant gating strategies was 0.02, 0.03, 0.07, and 0.02 for donors 1 - 4 respectively. This result was even more dramatic when looking at the difference in the median reported percentages of Melan-A-specific cells between close and distant gating strategies: 0.13, 0.18, 0.06, and 0.07 for donors 1 - 4 respectively. Obviously, such big differences preclude direct quantitative comparison of results generated across institutions that use different gating styles. Thus, description of gating style or displaying at least one example of a truly representative result would be highly recommended for any publication of MULTIMER experiments in human clinical trials, and is likely to be crucial for harmonization of the gating strategy in multi-institutional analyses.

**Table 8:** Gating Style

MULTIMER	Donor	Gating Style	Median		Close	Distant
CMV pp65	1	Close	0.13	↓		
		Distant	0.10			
			
	2	Close	0.05	↓		
		Distant	0.02			
			
	3	Close	0.18	↓		
		Distant	0.12			
			
	4	Close	0.08	↓		
		Distant	0.06			

Melan-A	1	Close	0.18	↓		
		Distant	0.05			
			
	2	Close	0.26	↓		
		Distant	0.08			
			
	3	Close	0.13	↓		
		Distant	0.06			
			
	4	Close	0.09	↓		
		Distant	0.02			

Overall Results Stratified by Close and Distant Gating Style. *(left) *The gating style of the participants were classified as "close" or "distant" based on the gating strategy applied. The table outlines the median percentages of MULTIMER-positive CD8-positive cells for each donor-antigen combination stratified by subgroup for those experiments meeting all three criteria for a positive response. *(right) *The dot plots present two representative examples of "close" and "distant" gating styles and the influence on resulting frequencies for the CMV-pp65 MULTIMER (upper row) and Melan-A MULTIMER (lower row).

**Figure 3:**
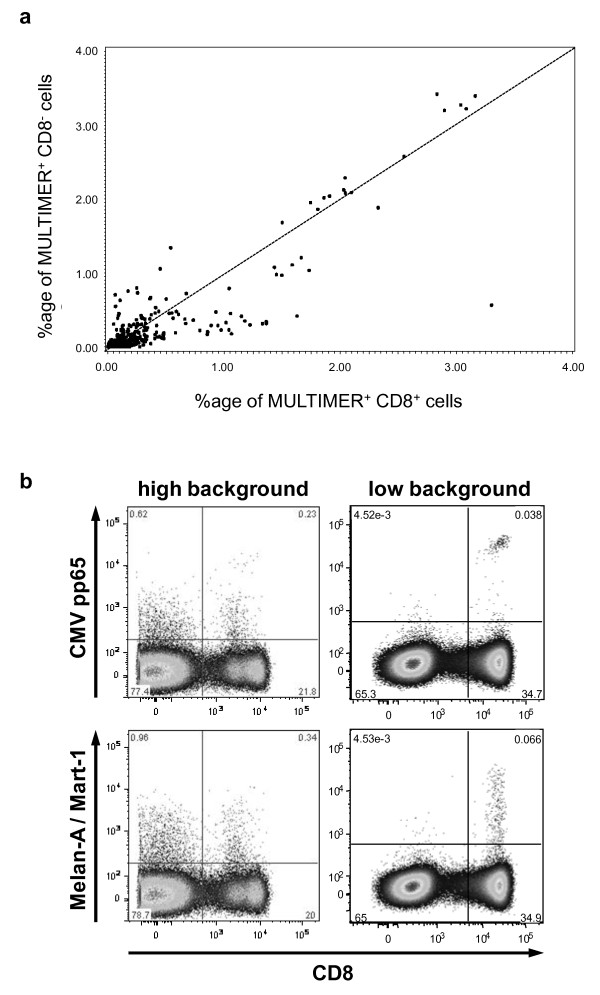
**MULTIMER binding to CD8-positive cells versus MULTIMER binding to CD8-negative cells**. (a) The Figure displays the percentage of MULTIMER binding to CD8-negative cells (y-axis) versus the percentage of MULTIMER binding to CD8-positive cells (x-axis) for each staining from a positive donor-antigen combination (DUMP and NO DUMP). (b) The four dot plots illustrate representative experiment results with a high background (left column) and a low background (right column) for the CMV-pp65 MULTIMER (upper row) and the Melan-A MULTIMER (lower row).

We further investigated whether binding of pp65 and Melan-A MULTIMERs in the CD8^+ ^versus the CD8^- ^compartment occurs independently. Figure 3a displays the percentage of MULTIMER binding in CD8-negative cells versus the percentage of MULTIMER binding in CD8-positive cells for each staining from all seven pp65- and Melan-A-positive donor-antigen combinations. The values of MULTIMER binding in CD8-positive and CD8-negative cells are linearly correlated (Spearman's correlation coefficient: 0.68, p < 0.001). The figure demonstrates that in dot plots where there is a large amount of MULTIMER staining in both CD8-positive and CD8-negative cells, the interpretation of the percentage of CD8+ MULTIMER positive cells might become questionable. Two representative examples are displayed in Figure 3b. Since MULTIMER-binding in the upper left and upper right quadrants does not always occur independently, we recommended that MULTIMER results be displayed in a way that enables the reader to determine the amount of MULTIMER binding in both the CD8-negative and CD8-positive cell fraction.

## Discussion

The results generated in this MULTIMER proficiency panel phase show that the introduction of a DUMP channel to a MULTIMER experiment on average will decrease the amount of non-specific MULTIMER-positive events in the CD8-cell population. The beneficial effects of applying a DUMP channel strategy were observed in non-censored data sets that employed laboratory-specific criteria for gating, as well as in a censored data set where a common strategy for excluded poor replicates and gating was employed. The reduction of non-specific MULTIMER-binding after introduction of a DUMP channel was observed in nearly half of all experiments performed (Figures 1a and 1b). Notably, we observed a 1.65-fold reduction of measured background MULTIMER-binding in the whole group with a large sub-group of experiments (approximately 50% of stainings) that showed a 4.1-fold median reduction of the background. The absolute median reduction in the fraction of experiments (48 of 100) that showed a clear decrease was 0.049% (about 1 in 2000 CD8 cells) and could be observed in protocols that used or did not use a DEAD cell dye. An *in silico *gating study showed a similar median background reduction for the independent use of DUMP channel markers and or dead cell dyes confirming the favorable effects of measures to gate out unwanted cells.

Although the observed differences might appear small, they can play a critical role. According to ICH guidelines (ICH Q2 (R1)) the background noise of an analytical test may be used to determine the lower limit of detection of an analytical test. Hence, measures to reduce background increase assay sensitivity. Consequently, the use of a DUMP channel and/or a dead cell marker can become essential to attain assay sensitivity in the range of 1 specific cell in 1,000-3,000 CD8^+ ^lymphocytes. Since most of the tumor antigen-specific CD8 T-cell responses, and also subdominant microbial specific CD8 T cells, are in this range, achieving a reliable sensitivity around this threshold value is central to establishing MULTIMER staining as a monitoring tool in translational immunological research [14,15]. The data sets generated in this proficiency panel phase suggests that in about half of all experiments performed in a variety of representative laboratories the detection of low frequency T-cell responses will not be technically feasible without use of a DUMP channel. In addition to increasing the test sensitivity, the use of DUMP channel antibodies may provide a more accurate measure of the true antigen-specific signal by decreasing the number of non-specific events in the CD8^+ ^cell population. Although use of a DUMP channel might lead to a reduced number of false-positive events in the quadrant displaying the MULTIMER-positive CD8-positive cells the only way to indeed confirm that a given event is a true positive signal would be to clone and functionally characterize the respective T cell or TCR.

A second outcome of this proficiency panel is that the use of intuitive filters for response determination can lead to an unexpected high number of experiments that will not be considered of being a successfully detected response. The organizers of this panel acknowledge that the cut-off value (200% difference) used to exclude inconsistent duplicates and the dot plot evaluation score were arbitrarily chosen and should not be considered as a standard strategy to filter results from MULTIMER experiments. The chosen filters should rather be seen as a pragmatic way to remove data sets that might include artefacts and to compute response detection rates to compare assay performance in the two tested conditions (DUMP vs. NO DUMP) of this proficiency panel. It is remarkable that although visual evaluation of dot plots is supposed to be highly subjective, disagreement between the central evaluation and the lab evaluation was only observed in 12% (74/636 stainings) of all collected dot plots. These results demonstrate that although visual inspection is a rather crude and highly subjective method for response determination, results generated across institutions lead to clearly discordant conclusions from a central evaluation only in the minority of cases. Although central optical evaluation of the dot plots can be a valid method to consistently rate data from MULTIMER experiments, the optical evaluation will always be inherently subjective. Hence there is an urgent need to develop algorithms and computer-based tools to identify clustered populations of events in a multi-dimensional data space which are under development [16-19]. Such algorithms could potentially lead to higher reproducibility, save time, and importantly, enhance gating strategies even for experienced operators.

The data shown in the third part of the results section (Table 8) clearly demonstrate that gating style can dramatically change the result of an experiment. Accordingly, we recommend adding at least one representative dot plot whenever results from MULTIMER experiments are published. This could be done either as part of the material and methods section or as supplementary electronic material and should enable better understanding of the experiment. This study also provided evidence that binding of MULTIMER to CD8^- ^and CD8^+ ^cells does not always occur independently of each other as suggested by the strong linear correlation shown in Figure 3a. Thus, we recommend that MULTIMER results be displayed in such a way that the investigator will also be able to view the amount of MULTIMER binding in the CD8-negative cell fraction.

Based on these results, we revisited the Harmonization Guidelines for MULTIMER experiments that were recently published [7]. Confirming the findings of the previous panel, the number of CD8^+ ^events acquired from the samples influenced the response detection rates. In experiments with less than an average of 100,000 positive CD8 cells counted, only 50% had a response detected. However, in experiments with more than 100,000 CD8 positive cells counted, 79% of all (including both pp65 and Melan-A) responses were detected (Additional file 1, Table S2). An additional confirmation of previous findings was that the use of more than 3 colors increased detection rates, compared to the use of only 2 or 3 colors (Additional file 1, Table S2). These findings confirm the relevance of the previously published harmonization guidelines.

## Conclusions

The main conclusion from this study is that use of a DUMP channel should be recommended whenever high sensitivity and accurate quantification of antigen-specific T cells is the primary goal. In addition our results suggest that the combination of a DUMP channel and a dead cell dye leads to the lowest non-specific MULTIMER binding observed after staining with an irrelevant MULTIMER with median values around 1 non-specific event per 5000 to 10000 gated CD8 T cells (Table 4). It has to be noted that the current proficiency panel design was not suited to formally determine the impact of a DEAD cell dye (no paired experimental data sets for this condition were generated) and thus the impact of using a DEAD cell dye needs further study.

**Figure 4:**
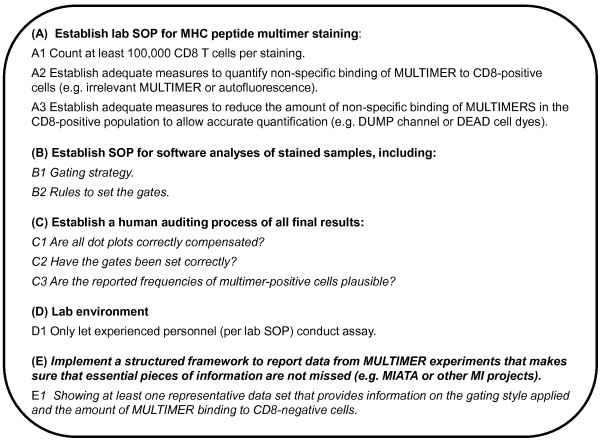
**Expanded CIC HLA-Peptide Multimer Harmonization Guidelines**.

The results generated in this panel confirm the harmonization guidelines from the first MULTIMER proficiency panel and necessitate the expansion of the existing guidelines for assay harmonization (Figure 4). The expanded harmonization guidelines include the recommendation to use irrelevant MULTIMERS to quantify the non-specific MULTIMER binding of the assay and to apply effective measures to keep the non-specific binding of MULTIMERS as low as possible. In addition recommendations on how to report experimental data from MULTIMER experiments could be deduced from this panel phase, including the request to provide sufficient information on the gating style and the amount of MULTIMER staining observed in by-standing CD8^-^.

## Competing interests

The authors declare that they have no competing interests.

## Authors' contributions

SA carried out the collection and assembly of data, performed data analysis, did the visual evaluation of all dot plots and wrote parts of the manuscript. LP coordinated the collection and assembly of data, did all statistical analysis and was involved in the interpretation of the data and manuscript writing. SJ did the overall project management, coordinated the distribution of material for the study, helped to interpret the data, wrote the manuscript and did the final approval of the manuscript. MK, MP, LMcN, TC, JY, KO and AH were driving the conception and design of the study, selected the donors for the study, interpreted the data and wrote the manuscript. PR was a co-leader of this study, and was involved in all activities starting from the concept phase until final interpretation of results and approval of the manuscript. He also coordinated the pre-testing experiments in his lab. CMB was the proficiency panel leader and mainly involved at all stages of the project, including organizational and scientific aspects, data analysis and interpretation as well as manuscript writing and approval. All authors read and approved the final manuscript. The members of the CRI-CIC Assay Working group critically reviewed and approved the study design prior to initiation of the study and critically commented to the final version of the manuscript.

## Supplementary Material

Additional file 1 Figure S1 and Tables S1 and S2 Click here for file
