# Supplementary material for: A critical assessment for the value of markers to gate-out undesired events in HLA-peptide multimer staining protocols
Source: J Transl Med. 2011 Jul 11;9:108. doi: 10.1186/1479-5876-9-108 (PMC3148571; doi:10.1186/1479-5876-9-108)
Supplement: Additional file 1 — Figure S1 and Tables S1 and S2 [file 1479-5876-9-108-S1.PPT]

## Slide 1
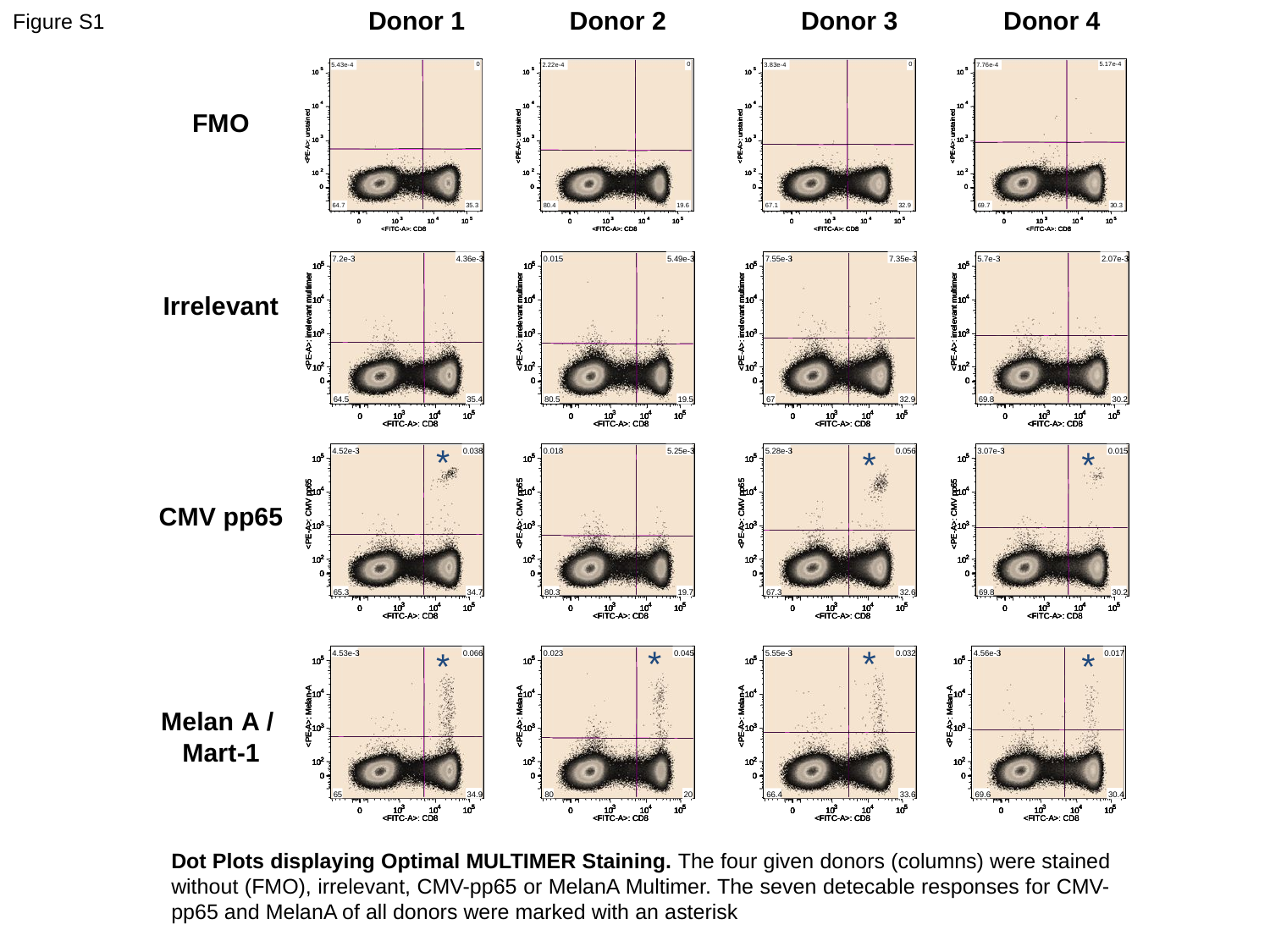

Figure S1
*
*
*
*
*
*
*
Dot Plots displaying Optimal MULTIMER Staining. The four given donors (columns) were stained without (FMO), irrelevant, CMV-pp65 or MelanA Multimer. The seven detecable responses for CMV-pp65 and MelanA of all donors were marked with an asterisk

## Slide 2
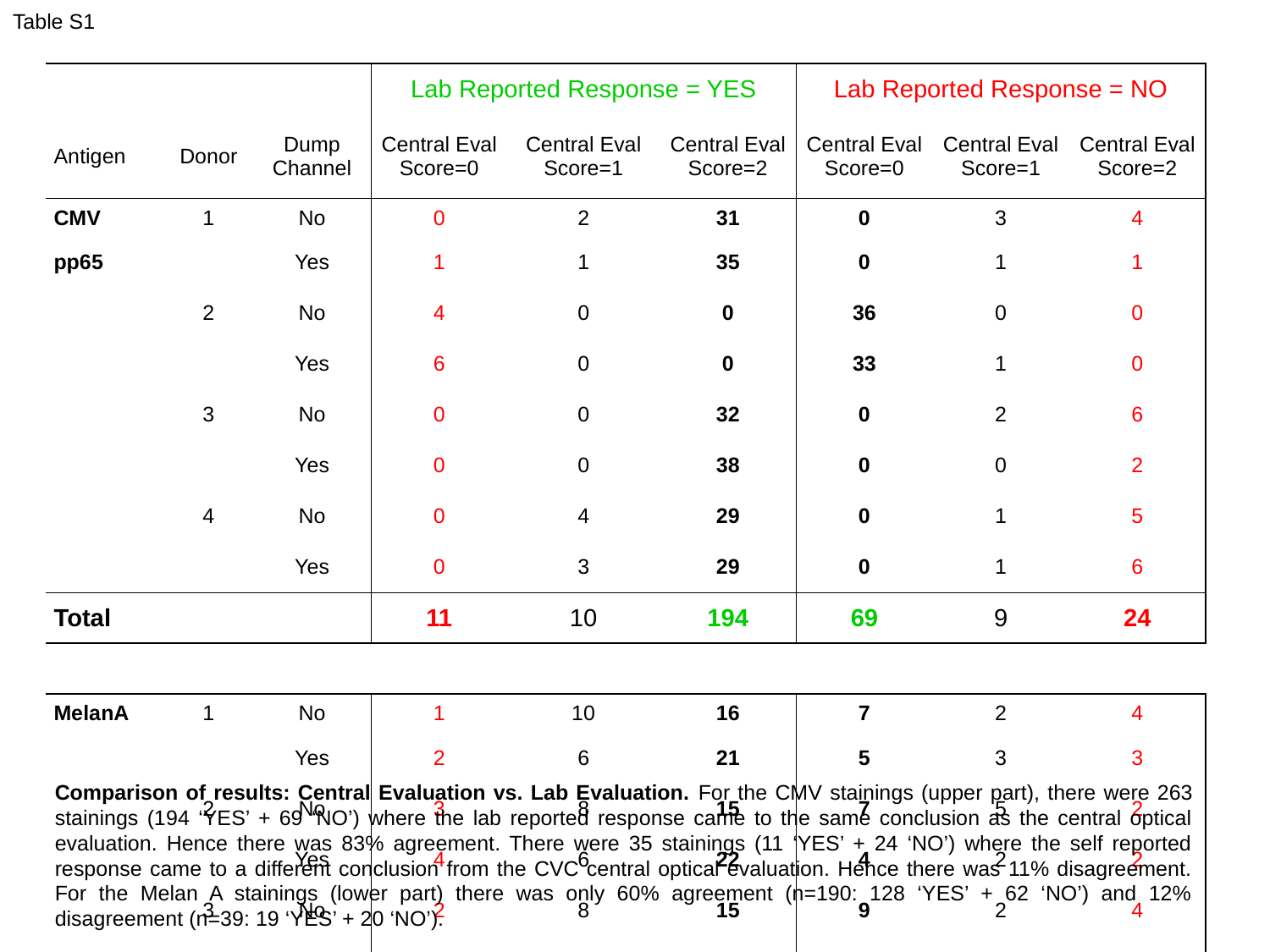

Table S1
| | | | Lab Reported Response = YES | | | Lab Reported Response = NO | | |
| --- | --- | --- | --- | --- | --- | --- | --- | --- |
| Antigen | Donor | Dump Channel | Central Eval Score=0 | Central Eval Score=1 | Central Eval Score=2 | Central Eval Score=0 | Central Eval Score=1 | Central Eval Score=2 |
| CMV | 1 | No | 0 | 2 | 31 | 0 | 3 | 4 |
| pp65 | | Yes | 1 | 1 | 35 | 0 | 1 | 1 |
| | 2 | No | 4 | 0 | 0 | 36 | 0 | 0 |
| | | Yes | 6 | 0 | 0 | 33 | 1 | 0 |
| | 3 | No | 0 | 0 | 32 | 0 | 2 | 6 |
| | | Yes | 0 | 0 | 38 | 0 | 0 | 2 |
| | 4 | No | 0 | 4 | 29 | 0 | 1 | 5 |
| | | Yes | 0 | 3 | 29 | 0 | 1 | 6 |
| Total | | | 11 | 10 | 194 | 69 | 9 | 24 |
| | | | | | | | | |
| MelanA | 1 | No | 1 | 10 | 16 | 7 | 2 | 4 |
| | | Yes | 2 | 6 | 21 | 5 | 3 | 3 |
| | 2 | No | 3 | 8 | 15 | 7 | 5 | 2 |
| | | Yes | 4 | 6 | 22 | 4 | 2 | 2 |
| | 3 | No | 2 | 8 | 15 | 9 | 2 | 4 |
| | | Yes | 1 | 8 | 20 | 3 | 5 | 3 |
| | 4 | No | 3 | 6 | 10 | 16 | 3 | 2 |
| | | Yes | 3 | 8 | 9 | 11 | 8 | 0 |
| Total | | | 19 | 60 | 128 | 62 | 30 | 20 |
Comparison of results: Central Evaluation vs. Lab Evaluation. For the CMV stainings (upper part), there were 263 stainings (194 ‘YES’ + 69 ‘NO’) where the lab reported response came to the same conclusion as the central optical evaluation. Hence there was 83% agreement. There were 35 stainings (11 ‘YES’ + 24 ‘NO’) where the self reported response came to a different conclusion from the CVC central optical evaluation. Hence there was 11% disagreement. For the Melan A stainings (lower part) there was only 60% agreement (n=190: 128 ‘YES’ + 62 ‘NO’) and 12% disagreement (n=39: 19 ‘YES’ + 20 ‘NO’).

## Slide 3
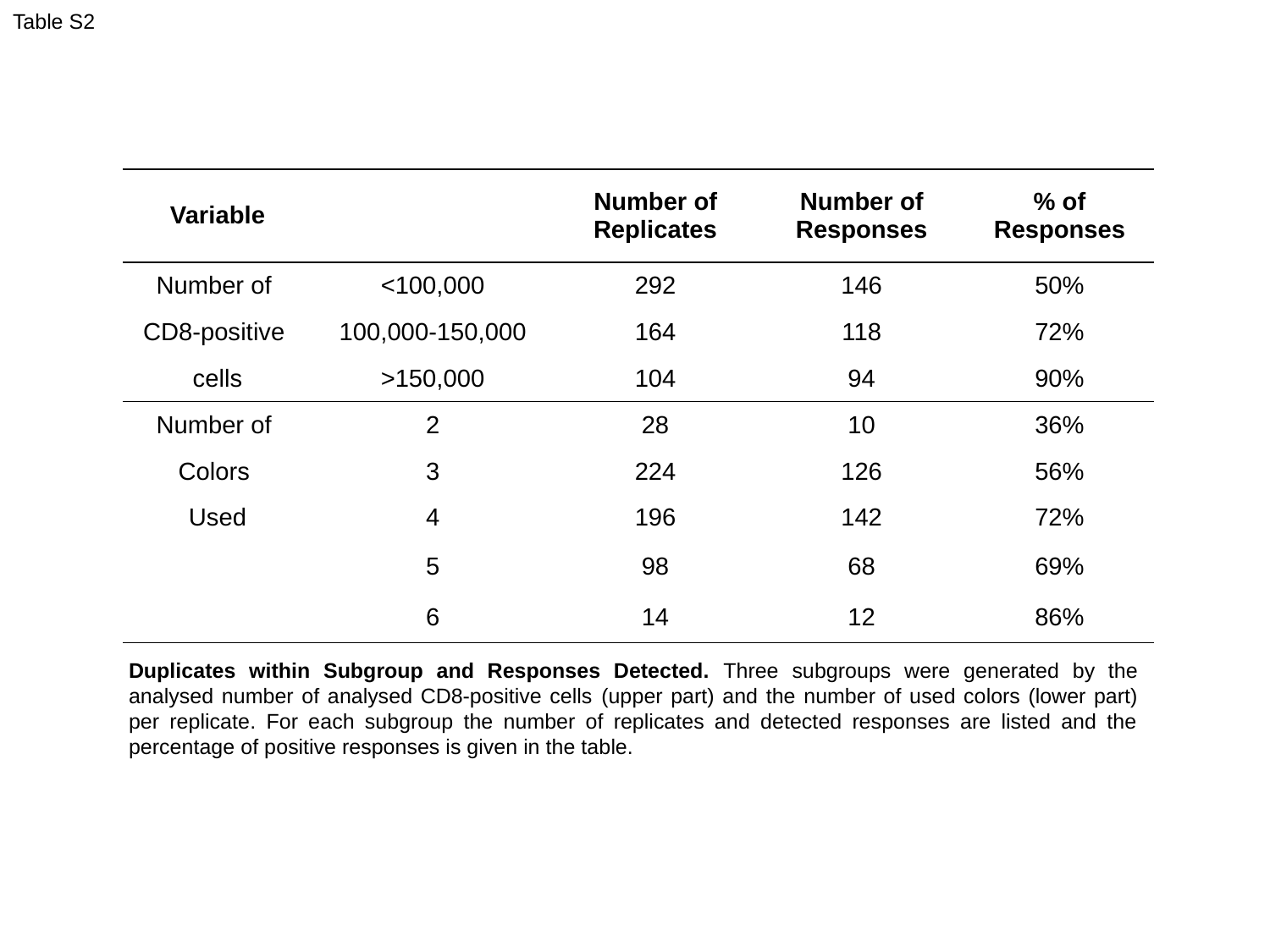

Table S2
| Variable | | Number of Replicates | Number of Responses | % of Responses |
| --- | --- | --- | --- | --- |
| Number of | <100,000 | 292 | 146 | 50% |
| CD8-positive | 100,000-150,000 | 164 | 118 | 72% |
| cells | >150,000 | 104 | 94 | 90% |
| Number of | 2 | 28 | 10 | 36% |
| Colors | 3 | 224 | 126 | 56% |
| Used | 4 | 196 | 142 | 72% |
| | 5 | 98 | 68 | 69% |
| | 6 | 14 | 12 | 86% |
Duplicates within Subgroup and Responses Detected. Three subgroups were generated by the analysed number of analysed CD8-positive cells (upper part) and the number of used colors (lower part) per replicate. For each subgroup the number of replicates and detected responses are listed and the percentage of positive responses is given in the table.
